# Neopeptide Analyser: A software tool for neopeptide discovery in proteomics data

**DOI:** 10.12688/wellcomeopenres.11275.1

**Published:** 2017-04-07

**Authors:** Mandy Peffers, Andrew R. Jones, Antony McCabe, James Anderson

**Affiliations:** 1 Institute of Ageing and Chronic Disease, University of Liverpool, Liverpool, L7 9TX, UK; 2 Institute of Integrative Biology, University of Liverpool, Liverpool, L69 7ZB, UK

**Keywords:** neopeptide, proteomics, mass spectrometry, extra-cellular matrix, biomarker, Progenesis QIP, semi-tryptic

## Abstract

Experiments involving mass spectrometry (MS)-based proteomics are widely used for analyses of connective tissues. Common examples include the use of relative quantification to identify differentially expressed peptides and proteins in cartilage and tendon. We are working on characterising so-called ‘neopeptides’, i.e. peptides formed due to native cleavage of proteins, for example under pathological conditions. Unlike peptides typically quantified in MS workflows due to the *in vitro *use of an enzyme such as trypsin, a neopeptide has at least one terminus that was not due to the use of trypsin in the workflow. The identification of neopeptides within these datasets is important in understanding disease pathology, and the development of antibodies that could be utilised as diagnostic biomarkers for diseases, such as osteoarthritis, and targets for novel treatments. Our previously described neopeptide data analysis workflow was laborious and was not amenable to robust statistical analysis, which reduced confidence in the neopeptides identified. To overcome this, we developed ‘Neopeptide Analyser’, a user friendly neopeptide analysis tool used in conjunction with label-free MS quantification tool Progenesis QIP for proteomics. Neopeptide Analyser filters data sourced from Progenesis QIP output to identify neopeptide sequences, as well as give the residues that are adjacent to the peptide in its corresponding protein sequence. It also produces normalised values for the neopeptide quantification values and uses these to perform statistical tests, which are also included in the output. Neopeptide Analyser is available as a Java application for Mac, Windows and Linux. The analysis features and ease of use encourages data exploration, which could aid the discovery of novel pathways in extracellular matrix degradation, the identification of potential biomarkers and as a tool to investigate matrix turnover. Neopeptide Analyser is available from https://github.com/PGB-LIV/neo-pep-tool/releases/.

## Introduction

Mass spectrometry (MS)-based proteomics can generate large amounts of data for downstream analyses, such as protein discovery, relative quantification and novel peptide fragment (neopeptide) discovery. The identification of neopeptides provides a platform for the development of antibodies that could assist in the discovery of molecular markers for diseases, such as osteoarthritis ^1^, as well as the identification of basic processes underlying disease, such as matrix turnover ^2^. Generating neopeptide antibodies enables the detection and monitoring of cartilage degeneration and therapeutic responses to treatment, in addition to providing treatment targets.

We have undertaken a number of studies to identify neopeptides following MS of ageing or diseased cartilage ^3^, tendon ^2, 4^, and synovial fluid ^5^, as well as following specific exogenous protease-driven digestion of cartilage extracts and in an *in vitro* model of early osteoarthritis ^1^. From these studies we have identified both novel and previously characterised neopeptides.

There are no available tools at present to interrogate the identified neopeptides. Therefore, in order to identify relevant neopeptides, we previously developed a novel LC-MS/MS data processing workflow. Under the previous workflow, we undertook “semi-trypsin” searches (i.e. only one terminus of the peptide was required to be the result of tryptic cleavage) with the relevant Uniprot databases using Mascot (Matrix Science, London, UK) or PEAKS Studio (Bioinformatics Solutions, Inc., Waterloo, Canada). The resulting identified peptides from individual samples were input into spreadsheets for further filtering. This data analysis was laborious and the inclusion of neopeptides to take forward user dependant. These factors inhibited users from generating neopeptide data with statistical confidence for further exploration.

To address this, we developed ‘Neopeptide Analyser’, a user-friendly interface for neopeptide discovery [in association with Progenesis QIP software for relative quantification (Waters, Manchester, UK)] that rapidly identifies neopeptides and provides a p-value to indicate differential expression. A key feature of Neopeptide Analyser is the ability to apply a statistical value to neopeptide discovery whilst also enabling the user to apply less stringent cut offs if required.

## Methods

The tool parses data files that are exported from Progenesis QIP (Version 2) ( http://www.nonlinear.com/progenesis/qi-for-proteomics/) in csv format. This is known as the peptide measurements csv file. The tool can also take a protein database (fasta) file as input, in order to search for the peptide locations. Two output files are produced by the tool. The first file is in the same format as the Progenesis QIP file, with the addition of three data columns (file input name suffixed ‘with_filter’ as default). For each peptide in the Progenesis output, these columns contain the residues preceding and following the peptide within its parent protein, and whether the peptide is fully tryptic (two termini resulting from trypsin digestion) or semi-tryptic (one terminus resulting from trypsin digestion). For some peptides, the sequence can be found in multiple proteins. For these cases, if the peptide could either be fully tryptic, or semi tryptic, it is assumed that the fully tryptic peptide is the most likely source.

The tool creates a second output file, also in csv format, which describes just the neopeptides that were found in the input file, and normalises the quantification values for each peptide (suffixed with ‘processed’. The normalisation method aims to remove the effect of changes in the overall parent protein abundance from the quantification value for the neopeptide, such that changes in abundance for the normalised neopeptide can be assumed to be the result of different extents of *in vivo* cleavage of the parent protein. Normalisation is thus achieved by dividing the candidate neopeptide (semi-tryptic) abundance by the sum of the abundance of all the tryptic peptides for the parent protein.

Where an experiment is setup with two conditions, these are read from the Progenesis QIP input file and a Student’s t-test is performed. This uses a normalised quantification value for each neopeptide, across the two conditions, and a p-value is produced so that the user can determine if the change in normalised abundance across the conditions may be significant. For discovery proteomics, this t-test may not be particularly meaningful by itself, as some peptides amongst many are likely to score a low value purely by random chance, so the output file also gives the Bonferroni (BF) corrected result, based on a user-supplied false discovery rate (FDR), as well as Benjamini-Hochberg (BH) corrected p-values. BH is generally the preferred method of global correction in quantitative proteomics, as BF is often too conservative to gain significance amongst large numbers of peptides/proteins.

### Implementation

The tool was developed as a Java application, with a 'Swing' graphical user interface (for compatibility with almost all desktop computers, requiring only a Java SE Version 7 Runtime environment installed, which has been available since 2011). An executable Java archive file can be downloaded, and opening this file will show the user interface.

The user interface allows the user to select a Progenesis QIP export file, as well as a fasta file to use as a protein database. These choices, and all other settings, are saved and restored each time the tool is started.

There are settings to allow the user to specify the format of the input file. The default settings are correct for files that are currently produced by Progenesis QIP, but these may change in future, or the data may have been manipulated in some other program (such as Microsoft Excel), before being used by the tool. The auto-detect features will usually be able to correctly identify the format of the file, by searching for columns that contain numerical data or only strings of amino-acids. The two output data files that are produced are given default names automatically based upon the input file name, but these can be changed via the user interface. The user can then click to process the input file, which will perform the computations needed to produce the two output files.

The default method for the tool is to search for the peptide in any matching proteins within the fasta file; it can then look at the previous and next residues in a matching protein, bearing in mind that the peptide could align with the C-terminus of the protein (and hence be fully tryptic even if it does not end with Arginine or Lysine). Similarly, if the peptide is at the N-terminus (peptide start position within the protein =1), the previous residue does not need to be tested, and where the previous residue is a Methionine (peptide start position =2), this is also not evidence of a non-tryptic cleavage, but N-terminal methionine cleavage, which is very common *in vivo*.

For large data files, a faster lookup method may also be employed, and can be selected from the options section. The database fasta file is used to build an internal dictionary of all possible tryptic peptides, including the required number of missed cleavages, which is determined by examining the input data file. The tool can then quickly see if any of the input peptides are in this fully-tryptic dictionary, or not, and even large files with many tens of thousands of peptides can be processed in less than a minute on a standard desktop computer. However, this method does not allow finding the preceding and following residues, as it is not feasible to create a dictionary of every possible semi-tryptic peptide.

In order to produce more meaningful statistical results, normalised neopeptide abundance values are created, and are included in the output file. The tool can detect two conditions that are present in the Progenesis QIP export file, and groups the sample data according to the relevant condition. It uses the calculated normalised quantification value for each neopeptide, in each sample, to produce a p-value, indicating the statistical significance of the variation across the two conditions (using Student’s t-test). The tool uses the FDR supplied by the user to output the result of BF correction, and follows the standard BH procedure for multiple testing to also give a corrected p-value in the output.

### Operation

The Neopeptide Analyser is available both as a pre-compiled Java executable file (NeopeptideTool.jar) and as java source code. No external libraries are used and the tool can be compiled with any compiler supporting Java SE version 7 or above. The pre-compiled Java executable file is compatible with any computer that has a Java runtime environment installed of version 7 or above. If it is not already installed, the runtime environment needed can be freely downloaded from https://java.com/en/download/.

## Use case

**Figure 1.**
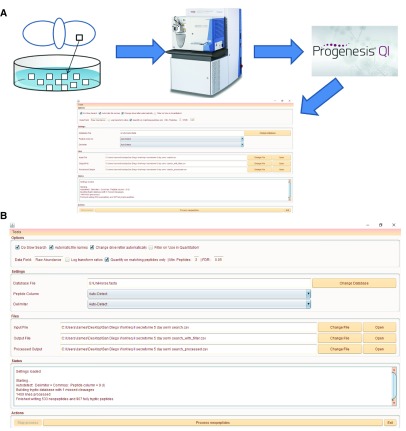
An example of Neopeptide Analyser workflow to analyse an equine cartilage secretome following treatment with IL-1β. ( **A**) Diagram of workflow incorporating liquid chromatography tandem mass spectrometry analysis with label-free quantification using Progenesis QIP and Neopeptide Analyser. In this example, cartilage explants are used from the metacarpophalageal joint of the horse and grown *in vitro* with and without IL-1β. ( **B**) Neopeptide Analyser interface showing file input and outputs. The data file used to generate this figure and Neopeptide Analyser output data files are available in Supplementary File 1 ( https://doi.org/10.6084/m9.figshare.4769746.v1 ^6^) and Supplementary File 2 ( https://doi.org/10.6084/m9.figshare.4772131.v1 ^7^). The ‘Options’ tab includes selection for slow search, ‘automatic file names’ (taken from input file name), drive letter selection, ‘use in quantitation’ filter, data field, ‘log transform ratios’, ‘quantify on matching peptides only’. Additionally, the minimum number of peptides and false discovery rate can be set manually. The ‘Settings’ tab enables the database file to be selected as well as peptide column and delimiter (default as auto-detect). The ‘Files’ tab contains options for the ‘Input File’, ‘Output File’ and ‘Processed Output File’. The ‘Status’ tab updates the user on the stage of analysis. Finally in the ‘Actions’ tab, the ‘Process neopeptides’ button is selected to start the analysis.

Figure 1 illustrates a typical use for Neopeptide Analyser. Data used for input were label-free quantification results following analysis of the secretome of equine metacarpohphalangeal cartilage explants treated with interleukin 1β for 5 days ^1^. Progenesis QIP was used to undertake label-free quantification of the proteins within the secretome following liquid chromatography tandem MS. Following feature picking, we exported the top three spectra for each feature. These were exported from Progenesis QIP and utilized for peptide identification with a locally implemented Mascot server in Unihorse database ( http://www.uniprot.org/uniprot/?query=equus%20caballus). Search parameters used were: 10 ppm peptide mass tolerance and 0.6 Da fragment mass tolerance; one missed cleavage allowed; fixed modification; carbamidomethylation; variable modifications; methionine oxidation and enzyme semitrypsin.

In Figure 1A the workflow of a typical experiment is demonstrated. Figure 1B details the input and output options on Neopeptide Analyser. The peptide measurement csv generated from Progenesis QIP was used as input (Supplementary File 1; https://doi.org/10.6084/m9.figshare.4769746.v1 ^6^). The Unihorse fasta file is applied to search for matching proteins. The two output files are evident as ‘Output file’ and ‘Processed output file’ (Supplementary File 2; ( https://doi.org/10.6084/m9.figshare.4772131.v1 ^7^). The processed output file details the protein, neopeptide sequence, preceding and following residues, p-value and FDR-adjusted p-value.

## Conclusions

Neopeptide Analyser enables rapid neopeptide detection from many thousands of peptides to be analysed within a minute using a standard computer. This will facilitate wider exploration of high-throughput proteomics data, leading to the identification of known neopeptides and the discovery of novel neopeptides. These may be used as indicators of matrix turnover, and as diagnostic or prognostic biomarkers. Whilst the tool enables the statistical significance of the variation across the conditions to be applied, the output enables the data to be interrogated with less stringent cut-offs that may be more applicable in some experiments.

## Data and software availability

The data input file for the Neopeptide Analyser is available in Supplementary File 1 in Figshare ( https://doi.org/10.6084/m9.figshare.4769746.v1 ^6^). The output and processed output files are available in Supplementary File 2 in Figshare ( https://doi.org/10.6084/m9.figshare.4772131.v1 ^7^).

Version 1.0 of Neopeptide Analyser is available to download from https://github.com/PGB-LIV/neo-pep-tool/releases/ both as a pre-compiled Java executable file (NeopeptideTool.jar) and as java source code.

Archived source code as at time of publication: https://doi.org/10.5281/zenodo.438664 ^8^

License: MIT
