# Supplementary material for: Neopeptide Analyser: A software tool for neopeptide discovery in proteomics data
Source: Wellcome Open Res. 2017 Apr 7;2:24. [Version 1] doi: 10.12688/wellcomeopenres.11275.1 (PMC5428739; doi:10.12688/wellcomeopenres.11275.1)
Supplement: Peer review history

# Peer review: Referee response for version 1

**DOI:** 10.21956/wellcomeopenres.12164.r22115

**Published:** 2017-05-10

**Authors:** Stephanie G. Dakin

**Affiliations:** 1 Nuffield Department of Orthopaedics, Rheumatology and Musculoskeletal Sciences (NDORMS), Nuffield Orthopaedic Centre, University of Oxford, Oxford, UK

In this manuscript, the authors develop a neopeptide analysis tool used that may be used in conjunction with label-free MS quantification tool Progenesis QIP for proteomics. Given the extensive use of MS based proteomics to analyse connective tissues in health and disease, and the difficulties associated with analyses of generated neopeptide data, the rationale for this study is clearly stated and the development of such a tool is timely. The findings from this study are likely to translate and be broadly applicable to MS analyses of other body tissues from human and animal species. The authors may wish to mention the potential broader utility of this tool in the manuscript. 

 In addition to the description of the software tool and details of the code, methods and analyses outlined in the manuscript, the authors may wish to provide material to support users that may encounter any problems during use of the software, for example a series of frequently asked questions or trouble shooting guide in the form of supplementary material.

I have read this submission. I believe that I have an appropriate level of expertise to confirm that it is of an acceptable scientific standard.

# Peer review: Referee response for version 1

**DOI:** 10.21956/wellcomeopenres.12164.r21632

**Published:** 2017-05-02

**Authors:** Timothy E. Hardingham, Jamie Soul

**Affiliations:** 1 Wellcome Trust Centre for Cell-Matrix Research, Faculty of Biology, Medicine and Health, University of Manchester, Manchester, UK

This paper presents the tool Neopeptide Analyser to detect neopeptides from proteomics data analysed with Progenesis QIP. The paper is well written and gives a clear explanation of the tools structure and application. There is no stand-alone documentation provided, but the tool was tested and found simple to use and ran quickly using the provided test data.

 Although dependant on the use of the Progenesis QIP software for the initial proteomic analysis, the purpose of the tool appeared well thought out to fulfil a specific need to detect unique peptides generated by different proteinases. It therefore appears to have strong potential for wide use to detect novel proteinase generated fragments and to perform differential quantification analysis of these neopeptides. Recent evidence suggests that different technology/software combinations generate some differences in results (e.g. Al Shweiki at al 2017), but results using this package are likely to be consistent and reproducible when using the same analysis format.

 The methods state that the output file also gives the Bonferroni (BF) corrected result, based on a user-supplied false discovery rate (FDR), as well as Benjamini-Hochberg (BH) corrected p-values, but only one adjusted p-value column is given in the output file?

 A sentence or two giving an example of the utility of the results would be useful and an example might be the differential analysis of fibronectin fragments.

We have read this submission. We believe that we have an appropriate level of expertise to confirm that it is of an acceptable scientific standard.
